# Cost of running a full-service receiving office at a centralised testing laboratory in South Africa

**DOI:** 10.4102/ajlm.v11i1.1504

**Published:** 2022-07-13

**Authors:** Naseem Cassim, Neeshan Ramdin, Sadhaseevan Moodly, Deborah K. Glencross

**Affiliations:** 1 Department of Molecular Medicine and Haematology, Faculty of Health Sciences, University of the Witwatersrand, Johannesburg, South Africa; 2 National Health Laboratory Service, Johannesburg, South Africa

**Keywords:** receiving office, cost per registration, order entry, costing, pre-analytical

## Abstract

**Background:**

The National Health Laboratory Service operates a platform of 226 laboratories across South Africa, ranging from highly sophisticated central academic hospitals to distant rural hospitals. The core function of the National Health Laboratory Service is to provide cost-effective and efficient health laboratory services in the public healthcare sector.

**Objective:**

This study aimed to assess the comprehensive cost of running a full-service receiving office (RO) at the Charlotte Maxeke Johannesburg Academic Hospital (CMJAH) laboratory.

**Methods:**

Top-down costing was conducted, with the cost per registration as the main outcome of interest. The annual equivalent costs (AEC) for the following categories were determined: registration materials, collection materials, staffing, laboratory equipment, building and electricity, and other operating costs. Data for the period from 01 April 2019 to 31 March 2020 were included in the analyses.

**Results:**

The AEC was $1 657 483.00 United States dollars (USD) and the cost per registration was $0.766 USD. Staff contributed 59.9% of the total cost per registration, while collection materials contributed 21.4%. The RO core staff (data clerks) contributed 50.8% of the total staffing costs, while messengers and drivers contributed 31.2%. The introduction of order entry at the CMJAH and other primary healthcare facilities reduced the total AEC by 20%. A single order entry application would serve both the CMJAH and primary healthcare facilities - hence we would prefer to not refer to order entries.

**Conclusion:**

Providing a comprehensive RO service costs approximately $1.00 USD per registration. The implementation of order entry at the CMJAH would reduce AECs substantially and improve efficiency.

## Introduction

The National Health Laboratory Service (NHLS) operates a platform of 226 laboratories across South Africa, ranging from central academic to distant rural laboratories, with a mandate to provide cost-effective and efficient services in the public healthcare sector.^1^ To carry out this mandate, the NHLS is premised on three pillars, namely diagnostics, research, and teaching and training.^1^

The Charlotte Maxeke Johannesburg Academic Hospital (CMJAH) is the largest NHLS reference laboratory in South Africa. The laboratory is International Standards Organization 15189-accredited and provides quality results that contribute to patient care.^2^ This laboratory houses the largest automated laboratory track system within the NHLS network that allows third-party instrument connectivity, thus providing a wide repertoire of clinical pathology services.^3^ The automated track creates a harmonious flow of samples through the pre-analytical, analytical and post-analytical phases and ensures that the laboratory can provide state-of-the-art diagnostic services with efficiency, improved turn-around times, and minimal wastage.^3^

An estimated 4.8 million tests are performed annually at the CMJAH laboratory, with steady year-on-year increases recorded. The receiving office (RO) plays a pivotal role in the correct and timeous capturing of samples into the laboratory information system (LIS) to facilitate the delivery of patients’ reports and expenditure reporting. Unfortunately, unlike the analytical phase at the CMJAH laboratory, the pre-analytical processes at the RO are not automated. Pre-analytics refer to the procedures carried out before actual sample testing and include patient identification, preparation, sample collection, sample packaging, sample transportation, and sample preparation for analysis and storage.^4^ Currently, the RO receives completed paper-based request forms to start the data capture process, which is time-consuming as it requires the entry of details of the health facility, healthcare worker, patient, and requested test into the LIS. Details such as the time of sample receipt, sample sorting, data capture, and delivery to the laboratory are also manually entered into the LIS. Furthermore, in the present system, samples are handled multiple times in the RO in contrast to the analytical phase where the automated track uses a one-touch approach.^3^

In other settings, many of these manual processes can be automated through order entry (OE). For example, public sector laboratories such as the Inkosi Albert Luthuli Central Hospital have adopted a paperless approach that uses OE to replace multiple manual RO tasks.^5^ Order entry is an application that enables healthcare workers to create electronic orders within the facility,^6^ replacing traditional pen-and-paper methods for ordering laboratory investigations using request forms. A simplified linear workflow for OE would be as follows: at source, the healthcare worker identifies the need for a laboratory test to be performed and uses the OE application to select the tests required, with the patient and healthcare worker information populated by an electronic patient record system (EPRC). The order is then electronically transcribed and sent to the national LIS where it is accepted and the testing process commences. After results authorisation, data are translated back to the EPRC and patient care is provided based on the results.^7^ With the OE workflow, all the required information is transmitted electronically and testing can commence with minimal RO processing, thus minimising transcription errors and improving order response,^8^ turn-around time, test ordering efficiency, laboratory utilisation, and, ultimately, patient care.^9,10^

As OE is part of the clinical workflow, it should ideally be implemented as a collaborative effort with the entire healthcare team.^7^ Order entry can influence and control test ordering patterns through structured order screens, manipulation of order sets and the analysis of real-time data to assess the impact of such changes.^10^ This is especially important in the context of national treatment guidelines such as for the care of HIV-positive patients, in which tests are performed based on standardised patient workup and care.^11^ Electronic gatekeeping, which is used to reduce unnecessary test requests, can also be programmed into the OE application to alert clinicians at the time of placing an order that their samples will likely not be processed.^11^

This study aimed to assess the comprehensive cost of running a full-service RO at a busy centralised academic laboratory that processes local samples and receives referred samples for specialist pathology testing and to assess the impact of the implementation of OE on RO costs.

## Methods

### Ethical considerations

Ethical clearance (approval number: M160978) was obtained from the University of the Witwatersrand Human Research Ethics Committee (Medical). No patient identifiers were collected and thus patient consent was not required.

### Study setting

This study was conducted at the full-service RO based at the CMJAH laboratory, Johannesburg, South Africa. Data are reported for the April 2019 to March 2020 financial period.

### Workflow at the Charlotte Maxeke Johannesburg Academic Hospital laboratory receiving office

Forty-two primary healthcare (PHC) facilities and two national central hospitals are directly served by the CMJAH laboratory; the laboratory offers all routine haematology, chemistry and microbiology testing, as well as specialist pathology testing (e.g. HIV viral load or CD4 testing, flow cytometry, cytogenetics, etc.). In addition, similar specialised tests that are referred from other PHC facilities and hospitals outside the immediate precinct of the CMJAH laboratory (*n* = 354) are also processed at the CMJAH laboratory, including those based in the West Rand, Ekurhuleni, City of Johannesburg, and Sedibeng districts.

**FIGURE 1:**
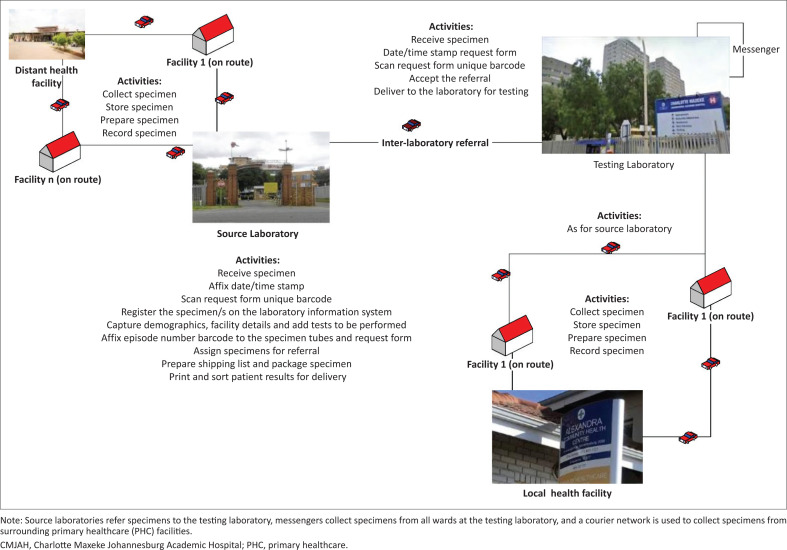
Sources of specimens and activities performed at the Charlotte Maxeke Johannesburg Academic Hospital laboratory receiving office, South Africa, April 2019 – March 2020.

Samples collected at distant health facilities are delivered to the closest local NHLS source laboratory using a hub-and-spoke courier network with multiple health facilities located along designated routes (Figure 1). These source laboratories serve their respective surrounding health facilities and perform routine clinical pathology testing. All specialised tests such as HIV viral load are subsequently referred within the NHLS network to academic testing facilities such as CMJAH. An inter-laboratory referral courier network is used to transport the referred samples to the larger academic laboratories. Locally sourced specimens are collected from hospital wards at CMJAH (*n* = 64; samples transported every 2 h) and the Nelson Mandela Children’s Hospital (*n* = 6; samples transported every 4 h). The final source of specimens is the surrounding health facilities (e.g. Alexandria Community Health Centre) that deliver samples directly to the CMJAH RO using a courier network.

Pre-analytical activities include sample receipt, sample sorting to identify where testing will take place, and registration on the LIS. These activities take place at both the CMJAH RO as well as at surrounding source laboratories. The registration process involves entering the details provided on the request form (patient demographics and health facility and healthcare worker details) into the LIS and attaching barcodes with the episode numbers on all samples. Samples not tested at the source laboratory are referred on the LIS; this involves preparing a shipping list and packaging samples for courier pick-up. The pre-analytical activities performed at the CMJAH RO are the same for surrounding health facilities. Referral samples are accepted on the LIS before testing. After testing, patient reports are printed and delivered to each health facility.

### Costing methodology

The costing analysis was done using Microsoft Excel (Microsoft Corporation, Redmond, Washington, United States). Costs were obtained using historical CMJAH expenditure data for the April 2019 to March 2020 financial period. The accounting stance was as a provider of the RO service, that is, all costs were obtained as the provider of laboratory services (costs incurred by the health facility were excluded). A top-down costing approach was used, with the main outcomes of interest being the annual equivalent cost (AEC) and cost per registration. The cost per registration refers to the costs of all the activities conducted in the RO for each patient visit, during which one or more tests may be requested. Therefore, it is not the cost per sample received as multi-disciplinary registration is performed. Multi-disciplinary registration refers to a single registration on a single episode number of multiple tests requested for multiple pathology disciplines. Costs were collected in South African rand (ZAR) and reported in United States dollars (USD) (using the monthly average exchange rate of R14.7835 ZAR to $1.00 USD for November 2019).^12^ The Consolidated Health Economic Evaluation Reporting Standards checklist was used in the preparation of this manuscript.^13^ Costs were reported for the following categories: registration materials, collection materials, staff, laboratory equipment, building and electricity, and other operating costs. The consumer price index ranged from 3.6% (November 2019) to 4.6% (February 2020).^14^ Furthermore, the World Bank reported an ‘Inflation, consumer prices (annual %)’ value of 4.12% for 2019.^15^ Therefore, we assumed a 4% discount rate. Organisational overhead costs were excluded; these included all corporate services such as human resources, finance, and information technology offered by the NHLS corporate offices.

Registration materials included items such as disposable gloves, N95 masks, thermal barcode printer paper, paper (to print worklists for the laboratory), disposal boxes, etc. Specimen collection materials included laboratory request forms, sharps disposal boxes, specimen collection kits and biohazard bags, which are all issued by the NHLS to healthcare facilities for sample collection.

Receiving office staff included the business manager (grade D5), the RO manager (D1), supervisors (C3), and operational staff, consisting of data clerks (B2/4), messengers (A3), administrative officers (C1), drivers (B4) and cleaners (A1). For staff costs, we used the annual salary data that include medical aid, pension and other allowances.^16^ Staff were categorised as RO core staff, messengers or drivers, RO management, cleaning, and overall management. The percentage of time spent on sample registration by each staff category was determined (Table 1).

**TABLE 1:** Percentage of time spent on registrations by staff categories and types at the Charlotte Maxeke Johannesburg Academic Hospital laboratory receiving office, South Africa, April 2019 – March 2020.

Staff category	Staff type	% Time spent on registrations
Messengers and drivers	Driver	100
Messengers and drivers	Messenger	100
Overall management	Business manager	20
Overall management	Secretary	20
Receiving office cleaning	Cleaner	100
Receiving office core staff	Data clerk	100
Receiving office core staff	Data clerk	100
Receiving office manager	LSS manager	100
Receiving office supervisor	Admin officer	100
Receiving office supervisor	LSS supervisor	100

LSS, Laboratory Support Services.

The costs of the following laboratory equipment were included: a messenger monitoring system (used to track and log the times when messengers arrive and collect samples at each ward and deliver samples back to the RO), date and time stamp devices, multi-function printers, barcode scanners, operator chairs, workbenches, computers, network points, barcode printers, specimen tube racks, shopping baskets (used for sample delivery to each section), air conditioners, fridges, freezers (–20 °C), document scanners, and filing cabinets. To calculate the AEC for laboratory equipment, working life was set at five years with a discount rate of 4%. Working life is defined as the period that an asset is likely to remain in service. We used the Microsoft Excel ‘sum’ and ‘PMT’ functions (equation 1):


sum (PMT(discount rate, working life, purchase price,0,1)). [Eqn 1]

Studies have reported that the ‘PMT’ function in Microsoft Excel is easy to use for calculating the AEC for equipment using assumptions of the discount rate, working life, and purchase price.^17,18^ To determine the total building costs, we multiplied the total area of the RO in square metres (m^2^) by the average building cost of R8163 ZAR per square metre.^19^ This average building cost is based on the Statistics South Africa estimate for building office spaces.^19^ To calculate the AEC for building costs, we used a working life of 50 years and a discount rate of 4% and applied this to the total costs using the PMT formula. We determined the monthly cost of electricity at the CMJAH laboratory and attributed 2% of the cost to the RO based on its size.

We used the RO income statement to assign other operating costs. These included the AEC for items such as cellular phones, computer consumables, printing, telephones, couriers, freight, postage, laundry, accreditation, waste disposal, uniforms, etc. The cost for LIS licenses and other costs were also obtained from the income statement.

### Costing analysis

We reported the AEC and the cost per registration. Data were reported for each cost category. The percentage contribution of each cost category to the total cost per registration was reported.

We assessed the impact of implementing OE at surrounding PHC facilities only versus implementing OE at all surrounding PHC facilities and all CMJAH wards. To calculate costs for each OE scenario, the percentage reduction in manual RO activities was applied to the staff costs of RO clerks. A rejection rate of 6% was used to determine the reduction in data collection materials and registration materials (based on LIS reports). Samples are rejected because of electronic gatekeeping rules (such as minimum re-testing intervals) or for failing to meet essential criteria stipulated in the laboratory handbook (such as missing mandatory information or using the incorrect specimen type).^20,21,22,23^ It was assumed that OE would integrate electronic gatekeeping and rejection rules to reduce wastage.^22,23^ By integrating these rules, the OE application would alert the healthcare worker that mandatory information was missing and prevent the order from being placed. This would reduce costs by preventing the use of specimen collection materials, as well as other time-consuming activities, including sample transport, sample sorting, data capture, and sample rejection. Currently, RO staff capture only 6% of samples that are rejected on the LIS. The AEC for the base case (as is) was compared to the two OE scenarios for each cost category.

## Results

Data from a total of 2 163 421 registrations at the CMJAH RO were included in the analyses. The CMJAH RO consists of 68 employees (excluding the business manager and administrative assistant), including 34 data capturers, 22 messengers, four drivers, four cleaners, three managers or supervisors, and one administrative officer. Assuming a 24/7 service for 365 days, this equates to a daily workload of 5927 registrations. On average, it takes 12 min per sample to complete sample receipt, sorting, registration, barcoding, and addition of test tubes to the track for delivery to each laboratory section.

### Annual equivalent cost

An AEC of $1 657 482 USD was reported for the CMJAH RO. Staffing contributed $992 664 USD (59.9%), collection materials contributed $355 450 USD (21.4%) and other operating costs contributed $222 653 USD (13.4%) to the AEC (Table 2). Registration materials ($54 622 USD), equipment ($20 444), and building and electricity ($11 650) collectively contributed $86 716 USD (5.2%) to the AEC.

**TABLE 2:** Annual equivalent costs and the cost per registration for each cost category at the Charlotte Maxeke Johannesburg Academic Hospital laboratory receiving office, South Africa, April 2019 – March 2020.

Category	Annual equivalent cost (USD)	% Annual equivalent cost	Cost per registration (USD)
**Cost category**			
Staff	$992 664	59.9	$0.459
Collection materials	$355 450	21.4	$0.164
Other operating costs	$222 653	13.4	$0.103
Registration materials	$54 622	3.3	$0.025
Equipment	$20 444	1.2	$0.009
Building and electricity	$11 650	0.7	$0.005
Total	$1 657 483	100.0	$0.766
**Staff category**			
Receiving Office core staff	$504 334	50.8	-
Messengers/Drivers	$309 718	31.2	-
Receiving Office Supervisor	$88 350	8.9	-
Receiving Office Manager	$54 389	5.5	-
Receiving Office Cleaning	$27 563	2.8	-
Overall management	$8309	0.8	-
Total	$992 664	100.0	-

USD, United States dollars; CMJAH, Charlotte Maxeke Johannesburg Academic Hospital.

### Cost per registration

The total cost per registration was $0.766 USD. Staffing contributed $0.459 USD, collection materials contributed $0.164 USD, other operating costs contributed $0.103 USD, registration materials contributed $0.025 USD, equipment contributed $0.009 USD, and building and electricity contributed $0.005 USD to the cost per registration.

### Staffing costs

The RO core staff (50.8%) and messengers and drivers (31.2%) contributed 82.0% of the total staffing costs, while the RO supervisor and RO manager contributed 14.4% of the total staffing costs.

### Impact of order entry

**FIGURE 2:**
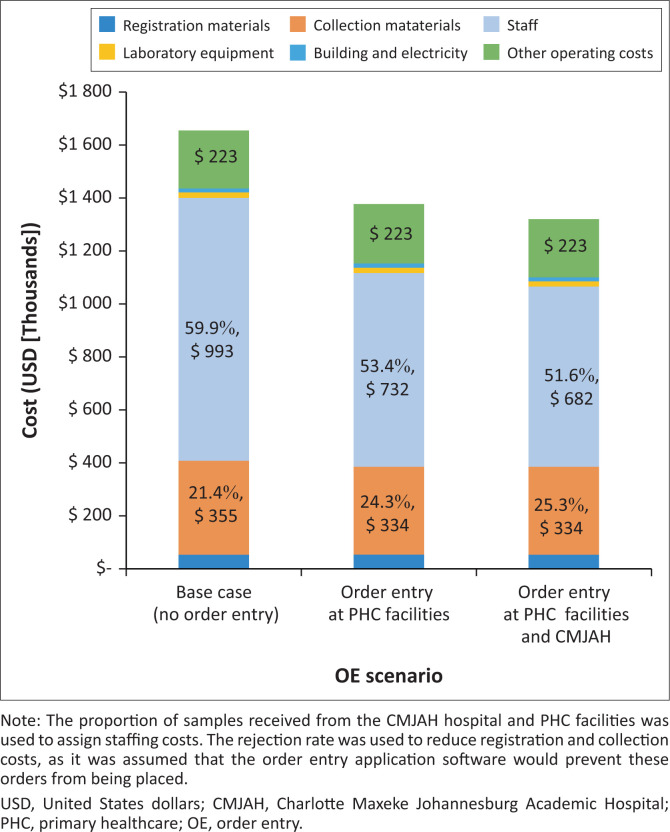
Impact of order entry implementation on the annual equivalent cost at the Charlotte Maxeke Johannesburg Academic Hospital (CMJAH) laboratory receiving office, South Africa, April 2019 – March 2020.

Introducing OE at the surrounding PHC facilities, which contribute 50% of registrations at CMJAH, would reduce costs by $285 081 USD. By implementing OE at the surrounding PHC facilities and the CMJAH wards, the estimated cost savings increased to $335 514 USD (Figure 2). This is a 20.2% cost reduction from the current costs of running the RO service.

## Discussion

The cost to register one sample at the CMJAH RO was less than $1.00 USD. Staffing was the major contributor to the overall cost, highlighting the manual nature of activities performed by RO staff. Collection materials and other operating costs, on the other hand, contributed only one-third of the total RO costs.

One of the current data challenges in healthcare services in South Africa is that patient demographics and personal identifiers are captured on multiple data systems in different formats. This highlights the inefficiencies of the current paper-based system. At the CMJAH, patients are assigned a unique patient identifier and their details are captured on the hospital information system when they first present for care. When they present to the pharmacy or radiology units, for example, their details are re-captured. Also, when a laboratory test is requested, this information is transcribed onto the laboratory request form and then manually captured in the LIS by data clerks. Aside from the multiplied workload, this multipronged, multi-layered approach to patient data capture and sample tracking can lead to transcription errors and data field omissions. These manual transcription errors have been confirmed during random data audits of patient request forms where differences were observed between LIS-contained information and information on the request forms (data not shown). Inefficient use of existing information within the healthcare system thus increases the workflow complexity and workload in the RO, thereby increasing the need for a large staffing component to process and capture information on the LIS.

For the implementation of OE across the public health sector in South Africa, the use of a national unique patient identifier is imperative to ensure consistency of information irrespective of the site of presentation. Currently, no unique patient identifier is used within the public health system.^24^ In the present system, duplicate patient records are inadvertently created on the hospital information system or EPRC systems as most PHC facilities use alphanumeric codes that are not based on the national health identifier (NHID) to identify patients.^25^ The development of an electronic system that can generate a master patient index or NHID would prevent unnecessary data duplication.^25^ This NHID can be implemented using a centralised, semi-distributed or highly distributed model.^25^ The different models determine where the NHID is assigned, that is, at the national, regional or facility level.^25^ The aim would be to develop an NHID system that contains a master record of all patients that have accessed public healthcare services across South Africa. A new NHID would only be assigned after searching the national master patient index list to prevent the duplication of patient information. This will facilitate the seamless data transfer of patient information to the OE application, enable longitudinal tracking of patients, and reduce healthcare costs by not repeating laboratory investigations that were already requested by another health facility.

The cost to implement OE (capital expenditure and maintenance) and an electronic health record system is a function of bed size.^26^ The one-time capital cost to implement OE for a 720-bed facility was estimated at $16 026 676 USD (2012 equivalent),^26,27^ and the annual maintenance costs were estimated to be $2 015 807 USD.^26,27^ As this data were reported for four hospitals with an existing electronic health record system, we calculated the cost for a single hospital and used the interest rates reported by the International Monetary Fund to determine the equivalent value in 2019.^28^ Between 2013 and 2019, the South African central bank policy rate reported a cumulative inflation rate of 6.63%.^28^ This equates to an annual cost of $4 809 449 USD per hospital in 2019.^28^ In contrast, a district hospital in Kenya reported a cost of $2.1 million USD for the implementation of OE and $435 000 USD for annual maintenance.^29^ Given that this hospital has 320 beds, this is not a reasonable cost estimate for OE implementation at CMJAH (1088 beds).^30^ Therefore, a local costing study is required to assess the implementation and maintenance costs of OE for all wards at CMJAH, Nelson Mandela Children’s Hospital, and surrounding PHC facilities.

Given the connectivity and information technology infrastructure challenges in the public health sector in South Africa, extensive investments would have to be made to make OE accessible and to develop the necessary interfaces with the different hospital information system and EPRC systems used by hospitals and PHC facilities. To implement OE at PHC facilities, some minimum infrastructures such as tablets, computers, Internet connection and an EPRC interface are required.^9^ These are required for the electronic transfer of orders to the LIS and the return of results.^9^ For this electronic transfer, logical observation identifier names and codes could be used.^31^

Implementing OE at the surrounding PHC facilities and CMJAH wards would result in an estimated annual cost saving of around 20%. Should the cost of OE installation be similar to the cost of the CMJAH RO, it would be possible to pay the capital cost of the system with the savings generated over a five-year period. There may also be additional cost savings generated by better adherence to treatment and pathology testing guidelines, reduced rejections and more appropriate ordering. Another study reported that the implementation of OE at a large academic hospital resulted in an annual saving of $2.2 million USD compared to an investment of $11.8 million USD, albeit it took over five years for these savings to be realised.^32^

As indicated by Birkmeyer et al., the primary motivation for introducing OE should be to improve the quality of care provided and not to reduce healthcare expenditure.^33^ It has been shown that OE has the potential to increase efficiency and effectiveness, thereby enhancing the quality of patient care.^32^ When implemented with clinical decision support systems, OE has the potential to reduce rejections and unnecessary test requests and alert clinicians of the required mandatory data fields and specimen criteria.^34^ In addition, as an electronic platform, OE could streamline the workflow in the health facility.

The development of EPRC systems in the United States has been challenging due to closed, proprietary and incompatible systems.^35^ Likewise, for developing countries, purchasing proprietary OE systems from developed countries leads to higher implementation costs due to travel costs and exchange rate fluctuation. As a result, the use of locally developed or open-source OE could result in lower implementation costs and minimal expenditure required to extend the application across the public health sector. A good example of a locally developed information system is Tier.Net, which is used to collect data on patients receiving antiretroviral therapy.^34^ After being piloted in the Western Cape province, Tier.Net is now used across South Africa.

**FIGURE 3:**
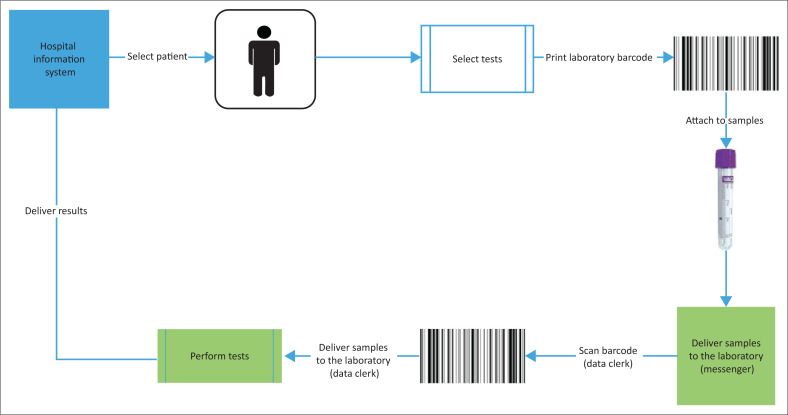
Typical workflow with order entry implemented at the Charlotte Maxeke Johannesburg Academic Hospital laboratory receiving office, South Africa, April 2019 – March 2020.

The implementation of OE across the public health system beyond CMJAH would dramatically streamline the process of placing laboratory orders and receiving results. The major benefit of OE would be a move from paper-based to electronic systems, removing the requirement to re-capture information that exists in the EPRC. In addition, the RO at laboratories would be dramatically scaled down given the electronic transfer of data. This would change the visibility of orders at both the health facility and laboratory, with better monitoring of samples and results that are outstanding. Overall, the interface with the laboratory would become efficient and streamlined by bypassing multiple manual steps in the current workflow (Figure 3).

Once OE is implemented, the CMJAH RO data clerks will not be retrenched. Staff may be re-allocated, deployed to other duties, or transferred to other laboratories with staff shortages. In addition, clerks could be encouraged to consider training as laboratory technicians and migrating to the analytical phase where shortages exist.

### Limitations

This study excluded the cost of organisational overheads. Should the organisational overheads be included, it would result in a minor change to the cost per registration as the organisational overheads would be divided by the number of registrations across the NHLS. The costs reported would also vary depending on the size and complexity of the RO, as well as the distance of the RO from source laboratories. In a small province such as Gauteng, health facilities are close (≤ 50 km) to testing laboratories. However, in more rural provinces such as the Northern Cape, facilities could be located over 300 km from their local laboratory, thus leading to higher logistics costs. Another limitation is that this study did not consider the costs of training required to introduce OE. Although the coronavirus disease 2019 pandemic has demonstrated that it is possible to conduct clinical training remotely,^36^ health facilities may not have access to the necessary bandwidth and computers present in a university environment.^36^ Therefore, the cost of introducing OE should be assessed given the challenges with remote learning, especially in rural settings.

### Conclusion

Providing a comprehensive RO service at a large referral laboratory in South Africa costs less than $1.00 USD per registration; however, most of this expenditure can be attributed to the high RO staffing costs due to the manual nature of RO activities. The implementation of OE has the potential to reduce RO costs by as much as 20% while also improving efficiency, reducing turn-around times, and improving patient outcomes.
